# What Does Economic Evaluation Mean in the Context of Children at the End of Their Life?

**DOI:** 10.3390/ijerph182111562

**Published:** 2021-11-03

**Authors:** Sebastian Hinde, Helen Weatherly, Gabriella Walker, Lorna K. Fraser

**Affiliations:** 1 Centre for Health Economics, University of York, York YO10 5DD, UK; helen.weatherly@york.ac.uk; 2 Yorkshire and Humber Children′s Palliative Care Network, Wetherby LS23 6TX, UK; gabriella.walker@nhs.net; 3 Family Advisory Board, Martin House Research Centre, York YO10 5DD, UK; 4 Martin House Research Centre, Department of Health Sciences, University of York, York YO10 5DD, UK; lorna.fraser@york.ac.uk

**Keywords:** end of life, palliative, paediatric, health economics, economic evaluation, cost effectiveness analysis, impact inventory

## Abstract

The ‘conventional framework’ of economic evaluation, the comparative public sector healthcare costs and quality adjusted life year (QALY) of two or more interventions, has become synonymous with commissioning decisions in many countries. However, while useful as a framework in guiding value-based decisions, it has limited relevance in areas such as end of life care in children and young people, where the costs fall across multiple stakeholders and QALY gains are not the primary outcome. This paper makes the case that the restricted relevance of the ‘conventional framework’ has contributed to the inconsistent and varied provision of care in this setting, and to the knock-on detrimental impact on children nearing the end of their lives as well as their families. We explore the challenges faced by those seeking to conduct economic evaluations in this setting alongside some potential solutions. We conclude that there is no magic bullet approach that will amalgamate the ‘conventional framework’ with the requirements of a meaningful economic evaluation in this setting. However, this does not imply a lack of need for the summation of the costs and outcomes of care able to inform decision makers, and that methods such as impact inventory analysis may facilitate increased flexibility in economic evaluations.

## 1. Introduction

Economic evaluation methods of cost-effectiveness analysis have become synonymous with the deliberative process of health technology agencies such as the National Institute for Health and Care Excellence (NICE) in England. An expression of the healthcare system costs and patient health benefits of an intervention compared to relevant alternatives, the approach’s primary strength is its facilitation of consistent and transparent decisions about the relative value of the intervention.

One of the major success stories of health economists in recent decades has been the creation and application of a methodological framework with which to assess healthcare interventions covering a diverse range of illnesses using an incremental cost-effectiveness approach. Extensive details of the framework are available elsewhere [1] but in brief it assesses competing interventions by their relative impact on long term patient quality adjusted life years (QALYs), weighed against the costs borne by the public healthcare system. In a budget constrained healthcare system, any additional cost burden is weighed against a nominal threshold of the value of what would have to be disinvested in to invest in the new intervention. Use of a conventional methodological framework supports consistent comparison not only between competing interventions but also between those in different treatment areas. This framework has been extensively adopted by health technology agencies around the world, including but not limited to NICE in England, the Institute for Quality and Efficiency in Health Care (IQWiG) in Germany, the Pharmaceutical Benefits Advisory Committee (PBAC) in Australia, and the Canadian Agency for Drugs and Technology in Health (CADTH). In this paper we will refer to this approach as ‘the conventional framework’.

However, the application of the conventional framework regarding what and whose health outcomes and cost burden matter has been to the detriment of the relevance of the method to some intervention areas, where the characterisation of benefit of care in terms of QALYs alone, or only a consideration of the costs to the healthcare system, is insufficient. A prime example of this is end of life and palliative care [2,3], where the characterisation of the decision problem goes beyond the patient and public healthcare system dynamic adopted in the conventional framework. In this setting, the use of standard QALYs to conceptualise the value of healthcare benefits is less clear as care is no longer targeted at curative treatment, but a wider range of dimensions of benefit, including comfort, feeling at peace, and family wellbeing among others, are excluded [4]. Previous authors have argued that the use of QALYs and the measuring of patient benefits only risks creating a perverse incentive for a myopic view of health when conducting an evaluation of the relative merits of competing interventions in this field [3,5,6,7]. In addition, from the cost side, resource use and care may extend beyond that which is funded through the healthcare budget alone and therefore omits evidence on the implications for other budgets and sectors.

The limitations of applying the conventional methodological framework to end of life care in a paediatric setting are arguably even greater. Additional complexities include how to quantify and value quality of life whilst dying or the quality of death, identifying what benefits count and by whom, and whether the death of a child is viewed differently by society than that of an adult [8]. This is compounded by the exclusion of the large burden of care and quality of life impacts pre- and post-death of the child which falls on those they are close to such as their parents, siblings, families, and the charitable sector, from the standard characterisation of health impact under the reference case [9], and the role of societal views on what is considered to be a ‘fair innings’ of life [10].

These factors, along with ethical concerns associated with the characterisation of costs and quality of life in this population, have inevitably played a role in the dearth of economic evaluations conducted in a paediatric end of life setting, with a recent systematic review finding only five published studies in palliative care, none in children [11]. In an English setting this is both compounded and demonstrated by the limited emphasis placed on cost per QALY in relevant policy discussions, including the current NICE Guidance on end of life care for children and young people [12]. This has led to a situation where economic evaluation is acknowledged as important in the commissioning of services in this setting [13], but rarely conducted and appearing to play scant role in policy deliberations [12]. This has resulted in there being almost no knowledge about the cost-effectiveness of services that are available to commissioners, and by extension about the quality of care provided.

The limited role of economic evaluation in this context is in many ways understandable, with the quantification of the health of children who are dying, weighing relative benefits against costs of care, being both complex [2] and potentially unpalatable [14]. Furthermore, the multi-stakeholder cost burden and large role of volunteers is outside of the conventional framework which focuses on the cost of healthcare only.

However, to exclude economic evaluation from the policy debate on this basis is to deny its potential role in effective decision making and commissioning. Furthermore, its exclusion erodes the role of accountability, demonstration of patient benefit, and the explicit consideration of the opportunity cost of investment, or lack of it, in the decision-making process [1]. These factors contribute to the inconsistent and varied provision of services which is evident in paediatric palliative and end of life care [15], and exactly the issues which agencies such as NICE was first created to address [16].

Previous literature has identified and reflected on this issue in adults [3,5,6,7,12,13,17], but has come to few conclusions, and there has been little in a paediatric setting. Therefore, in this paper we explore the challenges that face economic evaluations in this setting and for this population, consider how the published literature has sought to, or failed to, address them, and reflect on how the existing guidance produced by NICE in England, and recent methodological developments in incorporating multi-stakeholder objectives, can be applied to overcome some of these issues.

## 2. The Value of Economic Evaluation to the Decision-Making Process

Before we consider how economic evaluation can be used to best serve the children and young people who are nearing the end of their lives, those who are close to them, and the relevant clinical and commissioning decision makers, it is important to understand why economic evaluation as a discipline has emerged to play a useful role in many healthcare commissioning decisions.

At its core, economic evaluation concerns itself with the use of any finite resource, for example people, time, care, or money, in a transparent and consistent way to ensure that any decision that is made regarding investments in healthcare is considered explicit, defensible, fair and in keeping with what society considers appropriate [1]. Importantly, its role is not to usurp current decision makers or their current processes but to inform them [18].

The practical application of evaluations that address the needs of all relevant stakeholders while maintaining transparency and consistency is, however, a significant challenge, and one that has at its centre value judgements regarding whose costs and outcomes to consider, and how to measure and compare them. In a healthcare setting there are merits and limitations associated with any approach taken. At one extreme it is appealing to incorporate the costs and outcomes that fall on all those who bear any cost of an intervention and gain any health or wellbeing from it [19], often referred to as a “societal perspective”. Indeed, this approach may be in keeping with the desires of many stakeholders. However, such a broad framework suffers from the considerable task of collecting evidence from all stakeholders and determining how to compare different budgets and types of benefit [20]. Furthermore, in practical terms it is not clear who holds the responsibility as the societal decision maker nor the ultimate funding decision, given that in most settings there is no one supra-organisation whose remit covers all relevant interventions and areas of impact. At the other extreme, a simple description of the costs of relevant alternative interventions faced by the funder of the intervention in question, disaggregated by sector/funder, is very achievable but provides little with which to inform the choice about the relative merits of interventions across populations and sectors. It is within this latter case which economic evaluations of end of life care has primarily found itself [11].

The goal, therefore, is to find a balance whereby the costs and outcomes of enough stakeholders are included in a transparent and consistent way to produce an evaluation that is sufficiently informative to add value to the decision-making process. This balance should not place excessive demands on that evidence collection and analysis which is not worth conducting, nor apply weightings to the various stakeholders that are not indicative of societal preferences [21].

To fulfil the consistency requirement, it is important that any economic evaluations of services vying for the same finite healthcare resources use the same evaluation perspective. To compare two interventions competing for the same healthcare funding, even if indirectly, the same criteria must be applied to how to measure and value costs and effects, and which ones to exclude. This is not to say, however, that a broader approach to the problem cannot be applied simultaneously, only that care is needed to ensure apples are not being compared with pears.

Therefore, inevitably the application of ‘the conventional framework’ to inform decision making deliberations in health care has benefits and limitations. Its relative simplicity of application alongside existing clinical effectiveness studies, such as randomised control trials, and consistency with the decision-making problem faced by healthcare commissioners, whose primary focus is health maximisation and whose budgets are set exogenously, have led to wide-scale adoption. This has allowed agencies such as NICE to use it as a powerful tool to help it address issues such as the post-code lottery of pharmacological care that it originally set out to do, as well as consistency of decision making [16]. However, its exclusion of the financial impacts which fall beyond public healthcare providers, and outcomes other than patient health, creates an artificial boundary to the detriment of interventions such as end of life care which sit outside the conventional framework. The resultant system works well for pharmaceuticals and medical technologies but fails to reflect the complexities of life at the point of death and the plethora of preferences for care.

## 3. Challenges of Conducting Economic Evaluation in End of Life Care of Children and Young People

Debate over the best approach to address the challenges of conducting consistent evaluations in an end of life setting is long-standing, predating the routine application of the ‘conventional framework’ by health technology agencies. For example, in 1989 Goddard wrote, “This in part reflects the nature of services for the terminally ill where illness affects many aspects of the patients’ and their carers’ lives, and it is therefore difficult to condense the benefits to a single outcome measure, especially as measures such as life-years gained, often used in economic evaluation, would be entirely inappropriate for these patients. The problem with using a battery of measures is that it is difficult to then assess the overall effectiveness of different alternatives unless one care location dominates the other along all dimensions [22]”.

From a cost angle, while the public healthcare contribution to palliative and end of life costs is typically relatively straightforward to characterize [23], the care provided to children at the end of their life implies use of resources and a cost burden on a wider set of stakeholders, specifically charities, hospices, families, and the wider public sector [13]. As discussed earlier, under the ‘conventional framework’, these sit outside public healthcare and would not be included in the primary healthcare economic evaluation and yet have a direct impact on the care provided.

Regarding outcomes, both academic literature [14,24] and policy guidance [12,13] has reflected the limited role of a QALY-style outcome, which seeks to maximise quality of life over time, when curative treatment may no longer be possible or desirable. While avoiding the use of QALYs, in favour of specific end of life health outcomes, such as POS [24] and cPOS [25], is not an issue when conducting comparative cost-effectiveness analysis of relevant end of life alternatives, it becomes an issue when aiming to support consistent decision making across interventions, beyond end of life care, for example when disinvestment from elsewhere in the budget is needed to fund a more expensive end of life service [14]. Furthermore, many children who may be considered to be subject to end of life care may, given modern care developments, live beyond the short-term considered by such specific outcome tools.

These concerns are supported by the findings of Kinghorn and Coast [2] who through patient based qualitative research identified that while dimensions of pain, anxiety, and discomfort were addressed in the common conceptualisation of health in economic evaluations, many intermediate or process outcomes, such as planning and delivery of care and preparedness for death, were not.

Furthermore, given the age and dependency of this population (possibly requiring proxy valuation which has its own challenges), the impact of a child’s end of life, and therefore the value of high quality care, has significant and long reaching impacts on the families and those they are close to [9]. This is stated as an important consideration in the guidance on commissioning of end of life care [12,13]. This broader approach also contains analytic choices for which there are no clear answers, such as how many individuals to consider within the child’s network, as more potential benefits across different individuals would translate to higher benefits associated with that child and this would potentially disadvantage those children with a smaller network. Some authors have sought to address this much needed area of research, both through the development of outcome measures which incorporate the impact on the wider family, e.g., CPOS [25] and the Carer Experience Scale, but also through the consideration of how quality of life to the carer and the patient interact and can be compared [26].

More generally, the ‘conventional framework’ seeks to inform a single, centralised, publicly funded, healthcare commissioner with a defined healthcare budget. As has been discussed elsewhere [27,28] the relevance of this approach is degraded under a number of conditions, including when the commissioner straddles multiple budgets, care spans multiple commissioners, or is not wholly publicly funded. As with the characterisation of the relevant costs and outcomes, the example of care of children and young people at the end of their lives is an extreme realisation of these issues for a number of reasons. Most notably, the commissioning of paediatric palliative care is often diverse and complex. For example, in England the commissioning responsibility currently falls on Clinical Commissioning Groups (CCGs) (and, soon, Integrated Care Systems (ICSs)); however, many children’s social care services fall within local authority remits, with charities and hospices playing a vital role for many children at the end of their lives and their families. However, with typically less than a fifth of hospice funding coming from the Government in the UK [29], the commissioning reality is much more complicated than can neatly fit into the conventional characterisation of a single public commissioner.

## 4. What Approaches Have Been Taken in the Literature

The applied economic evaluation literature on end of life care in children and young people is limited, as is the wider quantitative research on interventions and services in this population. This is demonstrated by the systematic review by Mathew et al. of economic evaluation of palliative care models in any population [11], which identified over 12,000 articles, but found only five to be fully relevant to their analysis, and all of these in adult populations. Of the five studies found, the authors reported a wide variation in the outcomes assessed and the perspective of evaluation taken.

Mathew et al. identified that studies which only incorporated the cost component of an intervention were the most common approach in the setting, with 43 of 52 (83%) studies in this category. A focus on the direct cost alone was the approach also taken in the exemplar economic evaluations in the NICE guidance for end of life care for infants, children and young people [12] which consisted of two costing analyses.

In addition to being the method chosen in much of the applied literature, placing the focus primarily on the cost element of interventions has also been proposed in the methods research in this setting. For example, Diernberger et al. argued that the poor relevance of current economic evaluation frameworks to end of life care implies that, “The goal therefore should be to reduce the financial burden of care of the dying on the healthcare system without compromising the level of care or a person’s quality of life [17]”. However, such an approach implicitly excludes the potential to improve the quality of care provided through economic evaluation, but still necessitates the ability to observe the quality of care, in order to ensure its continued standard, in some consistent manner. Arguably if it is possible to measure the level of care sufficiently to ensure it is not eroded, and then it is eligible to be included as an outcome in a cost-effectiveness analysis, making this proposal of limited practical application.

One approach to the limitations of the conventional framework that has been proposed is the use of return on investment (ROI) type analysis, a cost-benefit methodology which seeks to quantify all of the costs and benefits of an intervention in terms of monetary value [20]. This is the approach taken in evaluations such as the 2021 evaluation of children’s hospices in Scotland [30]. While ROI analysis has strengths linked to its ability to present costs and outcomes to multiple stakeholders in a single estimate of total monetary value, the lack of a consistent methodological approach for its application undermines the requirement for consistency outlined earlier in this paper. This often results in an oversimplification of very complex cost and outcome dynamics which may not fully incorporate the societal value of the opportunity cost of the intervention under consideration [20]. This is demonstrated by the evaluation of children’s hospices in Scotland [30], which only considers that health and social care resource use which is avoided, and societal productivity gains, from carers able to work.

Additional to the practical attempts to quantify the costs and benefits of end of life care and apply a cost-effectiveness framework, some authors have proposed reassessing the fundamental approach taken to conducting economic evaluations in this area. For example, Coast supports the use of a ‘capabilities approach’ to evaluating end of life care [31], where it is the existence of choice and the option to receive a wide variety of care that matters, rather than the specific option that is taken.

## 5. The Policy Framework Applied by NICE

As discussed earlier in this paper, the ‘conventional framework’ applied in most economic evaluations focuses on the costs to the public healthcare system and the health benefits to the patient being treated. However, while it is this approach that forms the foundation of many health technology agency evaluations and guidance, the economic evaluation frameworks applied by policy bodies is often more flexible and not necessarily the same as the patient and public healthcare dynamic adopted in the ‘conventional framework’.

Taking NICE as an exemplar of an agency that produces evaluations covering a range of areas relevant to end of life care in children and young people, there are a number of important areas where this flexibility in the evaluative approach can be demonstrated. In their ‘Developing NICE guidelines: the manual’ [32], NICE outlines the economic evaluation reference cases it considers relevant depending on the nature of the intervention, the form of primary outcomes, and where the burden of funding falls. In brief the appropriate reference cases are stratified into three groups: (i) where funding is by the NHS and personal social services (PSS) with health outcomes, (ii) funded by the public sector with health and non-health outcomes, and (iii) funded by the public sector with a social care focus. The three reference cases vary across the type of economic evaluation, costs and outcomes considered relevant, ranging from a cost per QALY analysis consistent with the technology appraisal reference case [33] when costs are borne by the NHS and the outcome of interest is QALY maximisation, to a wide ranging perspective including non-health effects and costs falling on anyone in society, with outcomes being determined on a case-by-case basis. However, fundamental to all three is a requirement to record all of the relevant costs and outcomes that occur as a result of the intervention under investigation.

Additional to these reference cases, NICE has supplementary advice on the appraisal of cost-effectiveness of those interventions that are provided at the end of a patient’s life that have the potential to extend life [34]. While of limited relevance to children and young people at the end of their life, to whom treatment with curative intent is limited, the supplementary advice outlines that treatments which are expected to be life-extending can be given special dispensation to be deemed cost-effective at a higher incremental cost-effectiveness ratio (ICER), explicitly giving greater weight to QALY gains for these patients if such a treatment becomes available. While this approach addresses concerns about the additional value that society might place on extending life to those at the end of their life relative to other populations, it has been argued as doing nothing to overcome the fundamental limitations of the QALY as a measure in this population, due to the limited scope of the analytical perspective, or to be applicable only beyond a limited set of pharmacological interventions [35]. However, it highlights that there is flexibility in policy frameworks as to the relative weight placed on QALY gains in contrast to the idea that ‘a QALY is a QALY is a QALY’ [36], i.e., that all QALYs have equal weight. It is also important to reflect that the methodological guidance is in a state of continual evolution, with NICE currently undergoing an extensive consultation period regarding how to improve its current advice [37].

However, often the flexibility of the relevant reference case is not reflected in practice, even within research produced by NICE. For example, the guidance on end of life care for infants, children and young people with life-limiting conditions [12] contains two economic evaluations, 24/7 community nursing and telephone support, and rapid transfer to preferred place of care. However, in both cases only a costing analysis was conducted, which considered the costs falling on the NHS and personal social services, with the case made that the use of the QALY was of limited value as an outcome measure. In this regard the evaluations do not adhere to the NICE reference case for economic evaluation which requires some valuation of benefit to be weighed against the cost, as well as the non-public sector costs to be considered. The approach taken in these NICE evaluations is indicative of the wider published end of life care literature, with Mathew et al. showing that, of 52 economic evaluations, 43 were solely costing studies which did not consider any form of outcome [11].

## 6. Discussion

Over a period of more than 30 years, the methodological literature has been highlighting the challenges of conducting economic evaluations in an end of life setting [22]. Almost solely relating to an adult population, it has appropriately highlighted the challenges of conducting economic evaluations of healthcare interventions provided at the end of a patient’s life, not only with other interventions in the same setting, but across the wider public healthcare setting. As the use of a ‘conventional framework’ of cost-effectiveness analysis has become increasingly commonplace, the literature highlighting the framework’s limited applicability to the setting has become more widely acknowledged [3,12]. However, while possible solutions have been proposed [6,17,19] none have been routinely taken up by the applied literature or health technology assessment agencies such as NICE in England, with the majority of economic evaluations being limited to simple costing analyses [11] which are of limited value to policy makers.

It is hard to apportion the cause of the limited application of economic evaluation methodologies to the end of life setting, especially in children and young people, despite decades of discussion of the issue in the literature. It is likely that a plethora of factors have played a role, including the ethical difficulties that are even more profound in this setting than conventional healthcare, the personal and subjective nature of the outcomes, and the complexity of evaluating interventions which fall across multiple stakeholder budgets. Additionally, the success of the ‘conventional framework’ of economic evaluation to inform policy deliberations may have had the effect of making ‘perfect the enemy of good’, such that the unsuitability of what is seen as the gold standard framework to end of life care has resulted in a reluctance to apply another approach, instead resulting in costing analyses alone [11].

While it has become clear through the applied and methodological literature that there is unlikely to be a magic bullet framework which overcomes the challenges of the setting while maintaining the consistency and transparency requirements of robust economic evaluations, it would be a mistake to assume, as the lack of applied literature appears to indicate, that there is no role for outcome based economic evaluations in an end of life care setting. In this respect it is important to reflect on the role of economic evaluations. The success of the ‘conventional framework’ and its integral role in NICE recommendations, especially regarding healthcare technologies, has led to its depiction as a hurdle needing to be overcome [38]. However, as argued by Williams, the role of economic evaluation, and in particular the QALY, is not to usurp the decision maker but to inform them, and ‘make explicit what might otherwise remain hidden’ [18], and therefore should aim to provide guidance rather than a binary recommendation to commissioners.

In this regard, it is vital that a middle ground is found for the evaluation of end of life interventions that provides additional evidence to inform decisions in a consistent and transparent way. A likely candidate from the recent methodological literature is the use of an impact inventory approach [28]. This approach seeks to record the cost and outcomes which fall on all stakeholders, much like a societal cost-benefit or cost-consequence analysis, but with the important addition of presenting the opportunity cost of the additional cost burden falling on each stakeholder, in much the same way as the cost-effectiveness threshold does in the ‘conventional framework’. The approach accepts that the estimation of a single metric of cost-effectiveness which incorporates and weighs all the stakeholder perspectives is impractical, and that the ultimate weighting is for the decision maker at the time to make. It instead presents all of the direct effects of each intervention, in terms of costs and outcomes, on each relevant stakeholder, as well as conceptualising the opportunity costs that fall elsewhere within that stakeholder’s remit, thus fulfilling Williams’ requirement that economic evaluation informs the democratic decision making process without usurping it [18].

Such an approach would be consistent with the NICE reference relevant to the end of life setting, and has the potential to significantly improve the consistency and transparency of current funding decisions around end of life care. However, research is still needed to determine the opportunity cost of spending in some sectors, and the approach requires significant data collection across multiple settings, challenges especially evident in the setting of paediatric palliative and end of life care where the existing funding and delivery of services is hugely complex and varied between areas. Furthermore, it is likely to be especially hard for commissioners to agree relative weighting of costs and outcomes across sectors in such an emotive topic.

While this analysis has been focused on a UK setting, primarily due to the developed nature of economic evaluation in informing policy deliberations in this setting, this is clearly a topic of international relevance. While challenging to review systematically, we do not believe any country which routinely employs economic evaluation to inform health policy takes an approach significantly different than that presented by NICE, with the recommendation of a more flexible but poorly defined approach in complex areas such as end of life care by agencies such as HAS in France [39] and IQWiG in Germany [40].

## 7. Conclusions

Failure to consider the costs or benefits of the range of end of life care packages in children has contributed to inconsistent provision of care throughout the NHS in England, and internationally. Furthermore, at a time of extensive budgetary pressures and rising numbers of children with life-limiting conditions [41] the inability to define the benefits of a healthcare budget or argue for the value of additional funding puts the delivery of end of life care on the back foot, with increasing reliance falling on third sector support, which itself is struggling under the burden [42]. However, the unsuitability of the ‘conventional framework’ of cost-effectiveness analysis and the potentially impossible challenge of identifying a framework, which weighs the different costs and outcomes falling across stakeholders into a single statement of cost-effectiveness [21,43], has led to what little applied research exists focusing on costing alone [11]. The resultant blind spot of research able to sufficiently inform policy deliberations directly impacts the children and their families who feel failed by the promises of universal healthcare, with a parent representative to this research commenting, ‘Something I always find useful to consider here is the principle of universal healthcare: To protect people from the catastrophic consequences/costs of ill health. Our NHS does not do that, as anyone with a disabled child will tell you’.

Methods such as impact inventory evaluation frameworks [28] may represent an important turning point in undertaking economic evaluations in end of life care, especially in children and young people. Concurrent with such methodological developments must be additional research into the scale and scope of the cost and outcome implications of the different forms of care that may be provided to children nearing the end of their life.

## 8. Glossary of Terms

### 8.1. Approaches to Economic Evaluation

Cost-effectiveness analysis (CEA)—interventions are compared in terms of their cost burden and outcome benefit, where outcomes are measured in a natural unit such as symptom free days or generalisable measures of health such as quality adjusted life years (QALYs). The intervention with the better level of beneficial outcome is considered the optimal strategy, subject to a consideration of the opportunity cost implied by any additional cost placed on the system. The aim of the analysis is therefore to ensure the greatest level of beneficial outcome given the budget restrictions of the commissioner.

Return on Investment (ROI)—a means of estimating the expected benefit of an intervention by comparing the financial costs of an intervention against the expected benefits, where the benefits are estimated in terms of their monetary value. The translation of the benefits into a financial value allows for the estimation of a ratio of benefit, where the expected benefit per unit of expenditure is summarised. These ratios can be compared across multiple interventions to determine the intervention with the best ROI per unit of expenditure.

Conventional framework—In the context of this paper we define the conventional framework as that where an evaluation seeks to maximise the lifetime health of the patient subject to the costs borne by the public health and social care systems. This framework conventionally takes a CEA approach using a QALY as the outcome measure of interest. The incremental cost per QALY ratio of competing interventions is contrasted with some measure of the opportunity cost of any additional cost burden that will fall elsewhere in the health and social care system, characterised as a cost-effectiveness threshold.

Capabilities approach—a framework that assumes the benefits of an intervention should not just be determined by what an individual does but also by the available options offered to them. In its simplest form the approach assigns additional value to the existence of choice to the individual.

Societal perspective—Where the conventional framework takes the perspective that it is the QALY impact on the patient that is being maximised subject to costs to the public health and social care sector, a societal perspective incorporates a wider definition of costs and benefits of relevance to the analysis. These wider definitions may include out of pocket costs, to the individual or to other public sectors, of non-QALY benefits both in terms of health, e.g., quality of a person’s death, and non-health, e.g., measures of education.

Impact inventory—an economic evaluation method which seeks to take a societal perspective by reporting the full range of costs and benefits of relevance when discussing the relative merits of competing interventions. The approach seeks to summarise the costs and benefits by sector, as well as the opportunity cost of additional expenditure relevant in each setting.

### 8.2. Outcome Estimation

Quality Adjusted Life Year (QALY)—a means of quantifying persons’ health or changes to it in terms of the length of life and the quality in which it is lived. The QALY value is a simple multiplication of the length of life by the quality, measured on a continuous scale where 0 is a quality of life so poor it is comparable to death and 1 is the best health imaginable.

EQ-5D—the most widely used generic set of measures of an individual’s health-related quality of life. Covering dimensions of mobility, self-care, usual activity, pain/discomfort, and anxiety/depression, the EQ-5D has three versions. The EQ-5D-3L is the historic measure, providing three levels of response for each dimension, the EQ-5D-5L sought to improve on the 3L by using five levels of response, and the EQ-5D-Y is a measure specifically designed for a paediatric population.

POS—the Palliative-care Outcome Scale is a range of measures for estimating the needs of patients undergoing palliative care and their families. The original POS (‘version 1’) was specifically designed to overcome the limitations of existing measures of the quality of the patient experience and to ensure a measure was available that reflected the holistic nature of patient and carer needs. The measure asks patients to score the severity of their symptoms in addition to other feelings such as anxiety (by their and their family), depression, feeling at peace, sharing of feelings, and being well informed.

Children’s POS (C-POS)—a questionnaire under development that aims to be a version of the POS questionnaire which is applicable to children affected by life-limiting and life-threatening conditions and their families.

## Data Availability

No appliable.

## References

[1] Drummond M.F., Sculpher M.J., Torrance G.W., O’Brien B.J., Stoddart G.L. (2015). Methods for the Economic Evaluation of Health Care Programmes.

[2] Kinghorn P., Coast J. (2019). Appropriate frameworks for economic evaluation of end of life care: A qualitative investigation with stakeholders. Palliat. Med..

[3] May P., Cassel J.B. (2018). Economic outcomes in palliative and end-of-life care: Current state of affairs. Ann. Palliat. Med..

[4] De Wolf-Linder S., Dawkins M., Wicks F., Pask S., Eagar K., Evans C.J., Higginson I.J., Murtagh F.E.M. (2019). Which outcome domains are important in palliative care and when? An international expert consensus workshop, using the nominal group technique. Palliat. Med..

[5] McBride T., Morton A., Nichols A., van Stolk C. (2011). Comparing the costs of alternative models of end-of-life care. J. Palliat. Care.

[6] Coast J. (2014). Strategies for the economic evaluation of end-of-life care: Making a case for the capability approach. Expert Rev. Pharmacoecon. Outcomes Res..

[7] Noyes J., Edwards R.T., Hastings R.P., Hain R., Totsika V., Bennett V., Hobson L., Davies G.R., Humphreys C., Devins M. (2013). Evidence-based planning and costing palliative care services for children: Novel multi-method epidemiological and economic exemplar. BMC Palliat. Care.

[8] Eiser C., Morse R. (2001). A review of measures of quality of life for children with chronic illness. Arch. Dis. Child..

[9] Rogers C.H., Floyd F.J., Seltzer M.M., Greenberg J., Hong J. (2008). Long-term effects of the death of a child on parents’ adjustment in midlife. J. Fam. Psychol..

[10] Williams A. (1997). Intergenerational Equity: An Exploration of the ‘Fair Innings’ Argument. Health Econ..

[11] Mathew C., Hsu A.T., Prentice M., Lawlor P., Kyeremanteng K., Tanuseputro P., Welch V. (2020). Economic evaluations of palliative care models: A systematic review. Palliat. Med..

[12] National Institute for Health and Care Excellence (2016). End of Life Care for Infants, Children and Young People with Life-Limiting Conditions: Planning and Management.

[13] Public Health England (2017). Cost-Effective Commissioning of End of Life Care: Understanding the Health Economics of Palliative and End of Life Care.

[14] Round J. (2012). Is a QALY still a QALY at the end of life?. J. Health Econ..

[15] Together for Short Lives (2018). A Guide to Children’s Palliative Care.

[16] National Institute for Health and Care Excellence (2019). NICE: 20 Years of Evidence-Based Decision Making. https://indepth.nice.org.uk/20-years-of-NICE/index.html.

[17] Diernberger K., Shinkins B., Hall P., Kaasa S., Fallon M. (2021). Incompatible: End-of-life care and health economics. BMJ Supportive Palliat. Care.

[18] Williams A. (1991). Is the QALY a Technical Solution to a Political Problem? Of Course Not!. Int. J. Health Serv..

[19] Jönsson B. (2009). Ten arguments for a societal perspective in the economic evaluation of medical innovations. Eur. J. Health Econ..

[20] Edwards T.R., McIntosh E. (2019). Applied Health Economics for Public Health Practice and Research.

[21] Baker R., Mason H., McHugh N., Donaldson C. (2021). Public values and plurality in health priority setting: What to do when people disagree and why we should care about reasons as well as choices. Soc. Sci. Med..

[22] Goddard M. (1989). The role of economics in the evaluation of hospice care. Health Policy.

[23] Normand C., May P. (2019). Measuring Cost-Effectiveness in Palliative Care. Textbook of Palliative Care.

[24] Dzingina D.M., McCrone P., Higginson I.J. (2017). Does the EQ-5D capture the concerns measured by the Palliative care Outcome Scale? Mapping the Palliative care Outcome Scale onto the EQ-5D using statistical methods. Palliat. Med..

[25] Children’s Palliative Care Outcome Scale (C-POS). https://www.kcl.ac.uk/cicelysaunders/research/outcome/pos/children’s-palliative-care-outcome-scale-c-pos.

[26] Dhanji N., Brouwer W., Donaldson C., Wittenberg E., Al-Janabi H. (2021). Estimating an exchange-rate between care-related and health-related quality of life outcomes for economic evaluation: An application of the wellbeing valuation method. Health Econ..

[27] Hinde S., Horsfield L., Bojke L., Richardson G. (2020). The Relevant Perspective of Economic Evaluations Informing Local Decision Makers: An Exploration in Weight Loss Services. Appl. Health Econ. Health Policy.

[28] Walker S., Griffin S., Asaria M., Tsuchiya A., Sculpher M. (2019). Striving for a Societal Perspective: A Framework for Economic Evaluations When Costs and Effects Fall on Multiple Sectors and Decision Makers. Appl. Health Econ. Health Policy.

[29] Hospice UK Facts and Figures: Funding for Hospices. https://www.hospiceuk.org/about-hospice-care/media-centre/facts-and-figures.

[30] Hanlon J., Hex N. (2021). Children’s Hospices across Scotland: Economic Evaluation of Hospice Services.

[31] Coast J. (2019). Assessing capability in economic evaluation: A life course approach?. Eur. J. Health Econ..

[32] National Institute for Health and Care Excellence (2014). Developing NICE Guidelines: The Manual.

[33] National Institute for Health and Care Excellence (2013). Guide to the Methods of Technology Appraisal 2013.

[34] National Institute for Health and Care Excellence (2013). Appraising Life-Extending, End of Life Treatments.

[35] Bovenberg J., Penton H., Buyukkaramikli N. (2021). 10 Years of End-of-Life Criteria in the United Kingdom. Value Health.

[36] Weinstein M.C. (1988). A QALY is a QALY is a QALY—Or is it?. J. Health Econ..

[37] National Institute for Health and Care Excellence (2021). Methods, Processes and Topic Selection for Health Technology Evaluation: Proposals for Change.

[38] Rawlins M.D. (2012). Crossing the fourth hurdle. Br. J. Clin. Pharmacol..

[39] HAS (2020). ÉVALUER Choices in Methods for Economic Evaluation—HAS.

[40] IQWiG (2020). General Methods.

[41] Pinney A. (2017). Understanding the Needs of Disabled Children with Complex Needs or Life-Limiting Conditions.

[42] Together for Short Lives (2016). On the Brink: A Crisis in Children’s Palliative Care Funding in England.

[43] McHugh N., Baker R.M., Mason H., Williamson L., van Exel J., Deogaonkar R., Collins M., Donaldson C. (2015). Extending life for people with a terminal illness: A moral right and an expensive death? Exploring societal perspectives. BMC Med. Ethics.

